# Effects of Bone Marrow Mesenchymal Stromal Cell Therapy in Experimental Cutaneous Leishmaniasis in BALB/c Mice Induced by *Leishmania amazonensis*

**DOI:** 10.3389/fimmu.2017.00893

**Published:** 2017-08-10

**Authors:** Joyce Carvalho Pereira, Tadeu Diniz Ramos, Johnatas Dutra Silva, Mirian França de Mello, Juliana Elena Silveira Pratti, Alessandra Marcia da Fonseca-Martins, Luan Firmino-Cruz, Jamil Zola Kitoko, Suzana Passos Chaves, Daniel Claudio De Oliveira Gomes, Bruno Lourenço Diaz, Patricia R. M. Rocco, Herbert Leonel de Matos Guedes

**Affiliations:** ^1^Laboratório de Inflamação, Instituto de Biofísica Carlos Chagas Filho, Universidade Federal do Rio de Janeiro, Rio de Janeiro, Brazil; ^2^Laboratório de Investigação Pulmonar, Instituto de Biofísica Carlos Chagas Filho, Universidade Federal do Rio de Janeiro, Rio de Janeiro, Brazil; ^3^Laboratório Integrado de Imunoparasitologia, Campus Macaé – Universidade Federal do Rio de Janeiro, Macaé, Brazil; ^4^Laboratório de Imunobiologia, Núcleo de Doenças Infecciosas/Núcleo de Biotecnologia, Universidade Federal do Espírito Santo, Vitória, Brazil; ^5^Núcleo Multidisciplinar de Pesquisa UFRJ – Xerém em Biologia (NUMPEX-BIO), Polo Avançado de Xerém – Universidade Federal do Rio de Janeiro, Duque de Caxias, Brazil; ^6^Instituto Oswaldo Cruz, Fundação Oswaldo Cruz, Rio de Janeiro, Brazil

**Keywords:** Leishmaniasis, *Leishmania amazonensis*, cell therapy, mesenchymal stromal cell, BALB/c, IL-10

## Abstract

Cutaneous leishmaniasis remains both a public health and a therapeutic challenge. To date, no ideal therapy for cutaneous leishmaniasis has been identified, and no universally accepted therapeutic regimen and approved vaccines are available. Due to the mesenchymal stromal cell (MSC) immunomodulatory capacity, they have been applied in a wide variety of disorders, including infectious, inflammatory, and allergic diseases. We evaluated the potential effects of bone marrow MSC therapy in a murine model of cutaneous leishmaniasis. *In vitro*, coculture of infected macrophages with MSC increased parasite load on macrophages in comparison with controls (macrophages without MSCs). *In vivo*, BALB/c mice were infected with 2 × 10^6^ *Leishmania amazonensis* (Josefa strain) promastigotes in the footpad. 7 and 37 days after infection, animals were treated with 1 × 10^5^ MSCs, either intralesional (i.l.), i.e., in the same site of infection, or intravenously (i.v.), through the external jugular vein. Control animals received the same volume (50 µL) of phosphate-buffered saline by i.l. or i.v. routes. The lesion progression was assessed by its thickness measured by pachymetry. Forty-two days after infection, animals were euthanized and parasite burden in the footpad and in the draining lymph nodes was quantified by the limiting dilution assay (LDA), and spleen cells were phenotyped by flow cytometry. No significant difference was observed in lesion progression, regardless of the MSC route of administration. However, animals treated with i.v. MSCs presented a significant increase in parasite load in comparison with controls. On the other hand, no harmful effect due to MSCs i.l. administered was observed. The spleen cellular profile analysis showed an increase of IL-10 producing T CD4^+^ and TCD8^+^ cells in the spleen only in mice treated with i.v. MSC. The excessive production of IL-10 could be associated with the disease-aggravating effects of MSC therapy when intravenously administered. As a conclusion, in the current murine model of *L. amazonensis*-induced cutaneous disease, MSCs did not control the damage of cutaneous disease and, depending on the administration route, it could result in deleterious effects.

## Introduction

*Leishmania (Leishmania) amazonensis* is one of the etiological agents of the tegumentary leishmaniasis in Latin America (1) and occurs more frequently in the Amazon forest region (2). Infection with *Leishmania amazonensis* and other members of the Mexican *Leishmania* complex may lead to various clinical manifestations, and even with standard chemotherapy, some of them are relatively difficult to control (1). *L. amazonensis* is the etiological agent of cutaneous diffuse leishmaniasis (2), and in some cases, the infection may result in visceral leishmaniasis (3).

Infections with *L. amazonensis* differ from other *Leishmania* species infections due to their ability to suppress the activation and functions of immune cells, like macrophages, dendritic cells, and CD4^+^ T cells (4). Due to the susceptibility to infection with *L. amazonensis*, BALB/c mice have been used in several pre-clinical studies in order to understand the mechanisms of parasite’s evasion of the host immune defense system (5). To date, no ideal therapy for cutaneous leishmaniasis has been identified, and no universally accepted therapeutic regimen and approved vaccines are available. These limitations regarding an effective therapy highlight the need to develop new therapeutic strategies against cutaneous leishmaniasis.

Several studies have demonstrated that mesenchymal stromal cells (MSCs) can stimulate endogenous repair of injured tissues, modulate immune responses, and are recruited to inflammation sites through chemotactic mechanisms not completely understood (6). The MSCs promote repair and regeneration of damaged tissues by paracrine activity, through secretion of growth factors, cytokines, and chemokines. These factors can inhibit apoptosis, stimulate proliferation, promote vascularization, and modulate the immune response, promoting tissue regeneration (7). Stem cell therapy showed beneficial effects in cardiomyopathies (8, 9), including experimental chagasic cardiomyopathy caused by *Trypanosoma cruzi* protozoan (10–12). Therefore, since MSCs mitigate the progression of Chagas Disease (10–12), we hypothesized that it may also act on another parasites, such as *L. amazonensis*.

Leishmaniasis caused by *L. amazonensis* is an immunopathology with direct participation of CD4^+^ T cells in lesion development (13). Improving the lesion physiology due to the suppressive ability of MSCs to inhibit the formation of harmful effector T cell responses could be a strategy to treat leishmaniasis. There is no *in vivo* study evaluating the effect of MSC against leishmaniasis. Two different *in vitro* studies were performed using MSC, macrophages and *Leishmania major* to investigate phagocytosis. The first one demonstrated that macrophages pre-educated with MSC using trans-well system reduced phagocytosis of *L. major* (14). The other study from the same group demonstrated that cocultured macrophages with MSC pre-stimulated by soluble *L. major* antigens reduced yeast phagocytosis (15). However, the coculture of infected macrophage with MSC was not carried out.

In our work, we attempted to test the effects of bone marrow-derived MSC as an *in vitro* and *in vivo* therapy in a murine model of cutaneous leishmaniasis induced by *L. amazonensis*.

## Materials and Methods

### Animals

BALB/c mice originally obtained from the animal facilities of Universidade Federal Fluminense (UFF) were maintained in our own facilities with water and food *ad libitum*. Animals aged between 6 and 8 weeks were used. All experimental protocols used in this work were approved by the Ethical Committee for Experimental Animal Use established in the Instituto de Biofísica Carlos Chagas Filho—UFRJ under reference code CEUA IBCCF 157.

### Parasites

The parasites used in the experiments presented in this paper were *L. amazonensis* (MHOM/BR/00/Josefa strain) obtained by puncture of amastigotes from the lesion of infected BALB/c mice. The promastigotes were maintained at 26°C in M199 medium (Difco, NJ, USA) containing 10% fetal bovine serum (FBS; Gibco, NM, USA) until the fifth passage in culture to perform infection experiments. Infections were carried out with stationary phase culture, between the fourth and fifth days of culture.

### Preparation of MSC

Bone marrow cells were obtained from tibia and femurs of five BALB/c mice (20–25 g weight, with the age of 2 months), which were anesthetized with intravenous ketamine (25 mg/kg) and xylazine (2 mg/kg). Tissues were collected, rinsed with phosphate-buffered saline (PBS), transferred to a Petri dish, and cut into small pieces (approximately 0.2–0.8 cm^2^). The dissected pieces were washed with PBS, cut into smaller fragments, and subsequently digested with type I collagenase (1 mg/mL in DMEM/10 mM HEPES) for 30–40 min at 37°C. Any gross remnants that persisted after collagenase digestion were poured off between 1 and 3 min, and the supernatant was transferred to a new tube containing fresh medium and centrifuged at 400 × *g* for 10 min at 25°C. The pellets were resuspended in 3.5 mL Dulbecco’s Modified Eagle’s Medium (DMEM; Invitrogen, CA, USA) containing 1% penicillin and streptomycin, with concentration of 5,000 IU/mL and 5,000 µg/mL, respectively (Gibco, NM, USA), 20% of inactivated FBS (Invitrogen, CA, USA) and 15 mM HEPES (Sigma, MO, USA), seeded in T25 flasks (4 mL per flask) and incubated at 37°C in a humidified atmosphere containing 5% CO_2_. On day 3 of culture, the medium was changed, and non-adherent cells were removed. Adherent cells exhibited similar proliferation rates and, upon reaching 80% confluence, they were passaged with a 0.25% trypsin-EDTA solution (Gibco, NM, USA) and maintained in DMEM with 10% FBS (complete medium). Approximately 1 × 10^6^ cells were characterized as MSCs at the third passage according to the consensus of the International Society of Cell Therapy (16).

### Characterization of MSCs

Mesenchymal stromal cells were further characterized by their ability to differentiate into osteocytes, chondrocytes, and adipocytes (16, 17). Osteogenic differentiation was induced by culturing MSCs for up to 3 weeks in DMEM 10% FBS and 15 mM HEPES (Sigma, MO, USA), supplemented with 10^−5^ mM/L dexamethasone (Sigma, MO, USA), 5 µg/mL ascorbic acid 2-phosphate (Sigma, MO, USA), and 10 mM/L β-glycerolphosphate (Sigma MO, USA) (17). To observe calcium deposition, cultures were stained with Alizarin Red S (Nuclear, SP, Brazil). To induce adipogenic differentiation, MSCs were cultured with 10^−8^ M dexamethasone (Sigma, MO, USA), 2.5 µg/mL insulin, and 50 µg/mL indomethacin (Sigma, MO, USA) (18). Adipocytes were easily discerned from undifferentiated cells by phase-contrast microscopy. To further confirm their identity, cells were fixed with 4% paraformaldehyde with PBS and stained with Oil Red (Sigma, MO, USA) on day 21 of adipogenic differentiation. To induce chondrogenic differentiation, MSCs were cultured in DMEM supplemented with 10 ng/mL TGF-β1 (Sigma, MO, USA), 50 nM ascorbic acid 2-phosphate (Sigma, MO, USA), and 6.25 mg/mL insulin for 3 weeks. To confirm differentiation, cells were fixed with 4% paraformaldehyde in PBS for 1 h at RT and stained with Alcian Blue pH 2.5 (19) for detecting chondroblast secreted cell matrix (with chondrogenic differentiation) (data not shown).

Flow cytometry was performed using commercially available antibodies on FACSCalibur (Becton Dickinson, San Jose, CA, USA) following standard procedures. Cells were plated in 96-well plates and then centrifuged for 4 min at 300 × *g*, 4°C. Afterward, 10 µL of anti-CD16/32 antibody were added in cells (Fc receptor blocker), remaining for 15 min at 4°C. At the end of incubation, the cells were washed with PBS (100 µL/well), the plate was centrifuged at 300 × *g* for 4 min at 4°C and the supernatant was discarded. Subsequently, the cells were incubated with 10 µL of solution containing the anti-CD11b/FITC, anti-Sca-1/APC, anti-CD44/Pecy5, anti-CD45/PE, anti-CD49/PE, and anti-CD34/EFluor660 for 30 min at 4°C under light protection. Thereafter, the cells were washed with 100 µL of PBS and again centrifuged at 300 × *g* for 4 min at 4°C. After discarding the supernatant, the cells were resuspended in 200 µL of 1% PFA and transferred to FACS tubes containing 100 µL of PBS. The cells were analyzed using the FACSCalibur BD flow cytometry apparatus (BectonDickenson). Data were analyzed using FlowJo software 8.7 (Figures S1A–F in Supplementary Material). The respective antibody isotypes of the abovementioned antibodies or samples of unlabeled cells were used as controls. Undifferentiated population of MSCs was used in all experiments.

### Peritoneal Washing

Syringes containing 5 mL of RPMI (Gibco, NM, USA) medium were injected in the peritoneal cavity of BALB/c mice to obtain the macrophages. The medium containing the macrophages was withdrawn from the cavity and cells were counted with Tripan blue and transferred to a 24-well plate at the concentration of 5 × 10^5^ cells per well. The cells were incubated for 1 h at 37°C and 5% of CO_2_ for adhesion of macrophages to the plate. Thereafter, the plate was washed with warm PBS three consecutive times to remove the non-adherent cells, and then, 400 µL of RPMI with 10% of FBS supplemented with glutamine, pyruvate, and non-essential amino acids were added. After 24 h, the wells were washed again with PBS to remove B1 lymphocytes and 300 µL of RPMI with 10% of supplemented FBS were added.

### *In Vitro* Infection and Treatment of Infected Macrophages with MSC

The peritoneal cells were plated in a concentration of 5 × 10^5^ cells per well. The infection was performed with 2.5 × 10^6^ of *L. amazonensis* per well (ratio of 5:1) during 4 h with stationary promastigotes of *L. amazonensis*. After the infection time, the wells were washed with PBS and 5 × 10^4^ MSC were added to some wells and left for 48 h at 37°C and 5% of CO_2_. The plate was washed again with warm PBS three times. Finally, the plate was fixed and stained with a fast panoptic kit (Laborclin, RJ, Brazil). The infection was analyzed by optical microscopy (CX31, Olympus, Japan).

### *In Vivo* Infection and MSC Therapy

One week after being received from the UFF, the animals were infected by subcutaneous route with 20 µL of PBS containing 2 × 10^6^ promastigotes of *L. amazonensis* on the right rear footpad. 7 and 37 days after infection, animals were treated with 1 × 10^5^ MSCs, either intralesional (i.l.) (into the plantar cushion of the right rear footpad) or intravenously (i.v.), through the external jugular vein. Control animals received the same volume (50 µL) of PBS by i.l. or i.v. routes. MSCs (1 × 10^5^ cells) were administered in 50 µL by intravenous (i.v.) injection or 20 µL by intralesional (i.l.) injection. The animals were sedated with sevoflurane (Sevorane^®^, Abbot, IL, USA) for i.v. or i.l. administration.

### Clinical Profile (Lesion and Parasite Load)

The lesion growth was weekly monitored with a pachymeter in millimeter scale (Mitutoyo, Japan). The measurement of the uninfected footpad was then deducted. Forty-two days after infection, mice were euthanized and the infected footpad of each animal was removed, placed in a 70% alcohol solution for 1 min, and individually weighed. Additionally, the right popliteal lymph node was removed and placed in a vial with 1 mL of M199 medium. Footpads homogenates were obtained by manual maceration of the lesions with addition of 1 mL of M199 medium and, after sedimentation of the heavier particles, 10 µL of the supernatants were recovered and resuspended in 990 µL of medium, thereby obtaining a pre-dilution of 1:100. The number of parasites in the footpads, spleens, lymph nodes, and livers was determined by the limiting dilution assay (LDA). The macerates were diluted into 96-well culture plates (Jet Biofil, China) and incubated at 26°C for 15 days. Promastigotes cultures were observed with an optical microscope (Olympus, Japan) and the last well containing promastigotes was observed and noted (limiting dilution assay).

### Detection of Markers, Transcription Factor, and Intracellular Cytokines by Flow Cytometry Method (FACS) of T Cells

The cells of the macerate of the popliteal lymph node were quantified by counting in a Neubauer chamber (2 × 10^6^ cells per well in 24-well plates) to characterize T cells (CD4, CD8), regulatory T cells (CD4, CD25, FoxP3), and production of cytokines (IL-10, IFN-γ). The plates were centrifuged at 300 × *g* for 4 min. 100 µL of FACS buffer (PBS in 10% FBS) were added to the pellet and the cells were centrifuged again.

In the plate for characterization of regulatory T cells, non-specific-labeling blockage with anti-FCR (1:100) incubation was done for 5 min, followed by surface labeling with anti-CD4/Pecy7 (1:200) and anti-CD25/APC (1:200) for additional 30 min. The volume was made up to 200 µL in FACS buffer; the cells were centrifuged, followed by fixation in a kit fixation buffer (eBioscience) during 1 h. Then, the cells were washed and 100 µL of permeabilization buffer (eBioscience) kit were added to the pellet and incubated for 1 h, followed by centrifugation. Then, the cells were incubated with anti-FCR for 20 min and intracellular labeling was performed with anti-FoxP3/AlexaFluor 488 (1:50) incubating for 30 min. The cells were washed in permeabilization buffer and resuspended in FACS buffer for flow cytometry analysis (FACSCalibur system).

In the cytokine characterization plate, the cells were stimulated during 4 h with PMA (phorbol 12-myristate 13-acetate) (20 ng/mL), ionomycin (1000 ng/mL), and monensin (5 µL/mL) to inhibit cytokines secretion. Following the stimulation, the cells were washed in FACS buffer. Blockage of non-specific labeling with anti-FCR (1:100) incubation was done for 5 min, followed by surface labeling with anti-CD4/PeCy7 (1:200), anti-CD8/FITC for 30 min. The volume was filled to 200 µL in FACS buffer; the cells were centrifuged and further washed in FACS buffer. 100 µL of 1% paraformaldehyde was added to the pellet for fixation, which was left for 1 h incubation, followed by centrifugation. 100 µL of permeabilization buffer were added to the pellet (PBS and 0.1% saponin) and left for 1 h incubation, followed by centrifugation. The cells were incubated with anti-FCR for 5 min and then intracellular labeling was performed with anti-IL-10-PE (1:60) and anti-IFN-γ/APC (1:60) incubating for 30 min. The cells were washed in permeabilization buffer and resuspended in FACS buffer for flow cytometry (FACSCalibur system). The results were analyzed using software SUMMIT V4.3 Build 2445.

### Detection of Cytokines by the Enzyme-Linked Immunosorbent Assay (ELISA) Method

Concentrations of the cytokines present in the footpad homogenate supernatants were determined by the ELISA using commercial kits (BD OptEIA) according to the datasheet instructions. Recombinant murine IL-10, IL-4, and TGF-β were used to generate the standard curves. The detection limits for these tests are 31.3 pg/mL for TGF-beta and IL-10 and 15.72 pg/mL for IL-4.

### Statistical Analysis

No formal sample size calculation was performed to determine the numbers of animals per group; the sample size was based on the experience of our laboratory in previous studies using this model of cutaneous leishmaniasis (20). To compare *in vitro* data, Student’s *t*-test was used. Two-way ANOVA following Bonferroni’s *post hoc* test was used to compare all other parameters. *P*-values of 0.05 or less were considered meaningful. For *in vivo* experiments, we compared both groups, i.l. and i.v. This comparison between i.l. and i.v. was carried out besides of the differences between the two treatment protocols, according to the reviewer suggestion. All tests were analyzed using the statistical software package GraphPad Prism v6.0 (GraphPad Software, La Jolla, CA, USA).

## Results

### Characterization of Bone Marrow MSCs

Mesenchymal stromal cells adhered to plastic under standard tissue culture conditions and were able to differentiate into mesenchymal lineages, including osteocytes, chondrocytes, and adipocytes under *in vitro* conditions (data not shown). Additionally, MSCs expressed cell surface markers, such as: CD44, CD49 and Sca-1, bud did not express CD11b, CD45 and CD34 (6). We observed that in the third passage of the culture, most cells express CD44 (95%) (Figure S1A in Supplementary Material), CD49 (88.9%) (Figure S1B in Supplementary Material), and Sca-1 (91.2%) (Figure 1C in Supplementary Material) markers, and only a small fraction expressed CD11b (0.7%) (Figure S1D in Supplementary Material), CD45 (0.6%) (Figure S1E in Supplementary Material), and CD34 (0.4%) (Figure S1F in Supplementary Material) markers. Figure S1G in Supplementary Material represents the cell profile.

### Coculture with MSC Increased Parasite Load of Macrophages

**Figure 1:**
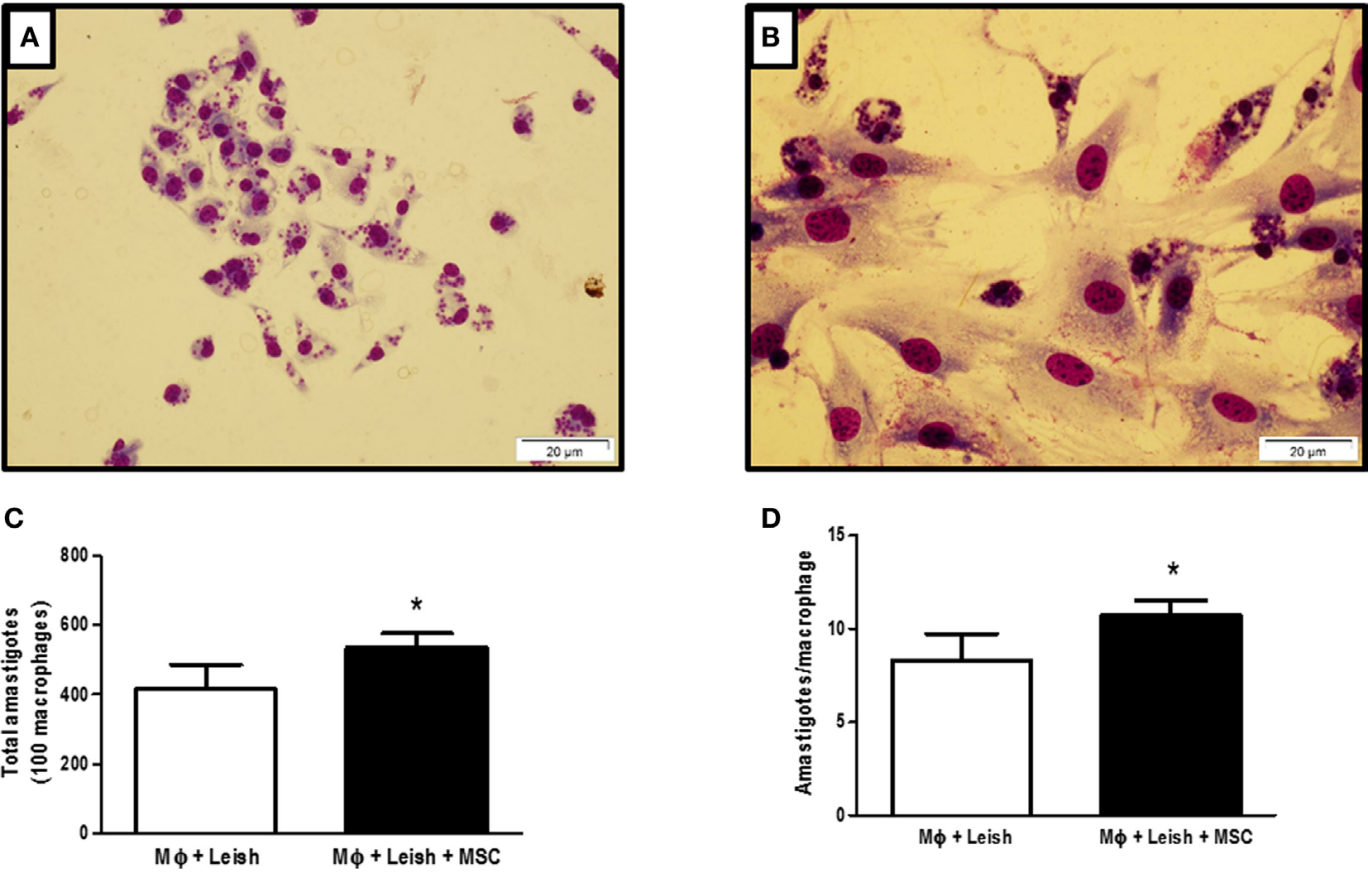
Macrophages infected with *Leishmania amazonensis*, cocultured with mesenchymal stromal cell (MSC) or not. **(A)** Macrophages infected with *L. amazonensis*; **(B)** coculture of infected macrophages with *L. amazonensis* and MSCs; **(C)** total number of amastigotes; **(D)** number of amastigotes per cell. Macrophages were isolated from peritoneal cavity and plated at 5 × 10^5^ cells per well. After 24 h cells were infected with 2,5 × 10^6^ *L. amazonensis* per well for 4 h and then washed. MSCs were plated at 1 × 10^4^ concentration per well. The plate was stained 48 h after the infection with fast panoptic kit. The *Leishmania* were counted on the optical microscope, Olympus CX31, at 100× magnification; Photo taken from inverted microscope Olympus BX51 at 40× magnification with the program Cell F 3.1. Fluorescence Microscope, OLYMPUS BX51. *N* = 1; triplicate average of one experiment (*P* ≤ 0.05). Results representative of three experiments.

We decided to evaluate the effect of MSC *in vitro* on infected macrophages using coculture of macrophages and MSCs. Figure 1A shows macrophages infected with *L. amazonensis* and Figure 1B shows macrophages infected with *L. amazonensis* in coculture with MSC. The presence of MSC increased the parasite load on macrophages *in vitro*, with a meaningful difference in terms of total number of amastigotes between infected macrophages (MΦ + Leish) and infected macrophages in coculture with MSC (MΦ + Leish + MSC) (Figure 1C). The same was observed regarding the total number of *Leishmania* by the amount of infected macrophages (Figure 1D). This result suggests that MSC induced an increase of susceptibility of the macrophages in the *in vitro* infection.

### Treatment with MSC by i.v. Route Showed a Non-Protective Effect on Infection by *L. amazonensis*

**Figure 2:**
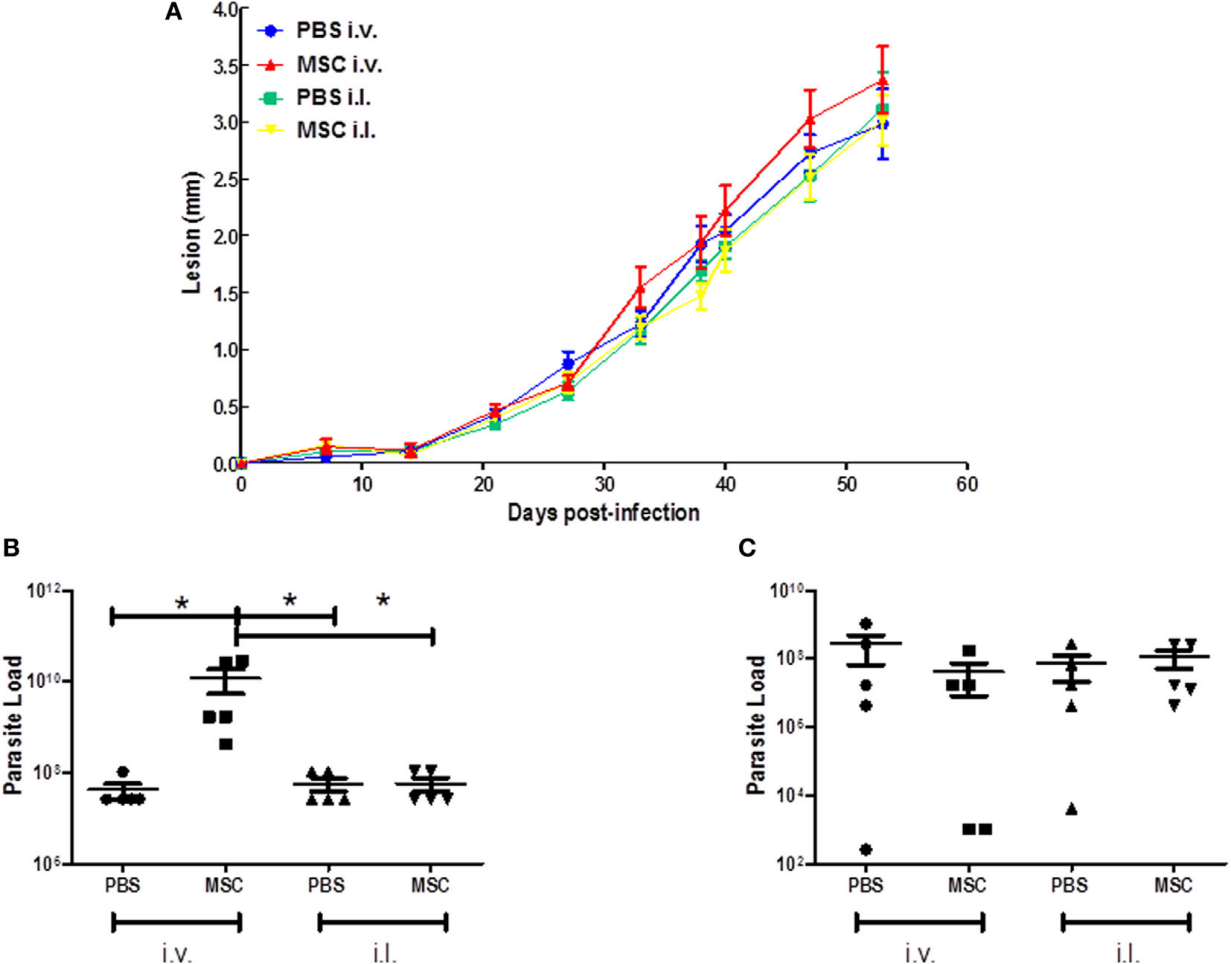
Effect of mesenchymal stromal cell (MSC) treatment on clinical profile. **(A)** Lesion growth during 42 days; **(B)** Parasite load of the footpads; **(C)** Parasite load of the popliteal lymph nodes. BALB/c mice were infected with 2 × 10^6^ of *Leishmania amazonensis* at the right footpad (day 0). At day 7 they received the first dose of treatment (1 × 10^5^ MSC cells). Animals from the MSC i.v. group were treated with cell injection (50 µL) in the external jugular vein. phosphate-buffered saline (PBS) i.v. animals received injection of PBS (50 µL) into the external jugular vein, while the MSC i.l. group was treated with cell injection (20 µL) at the same site of infection as so the PBS i.l. group, that received a injection (20 µL) of PBS. 30 days after the first dose (day 37), the second dose was applied in each group. The lesion growth in the footpad was measured weekly by pachymetry (in millimeter), discounting the measurement of the uninfected footpad. After 42 days, the animals were euthanized and footpads and popliteal lymph nodes were removed and macerated for the parasite load analysis by the limiting dilution assay (LDA) (*) *P* ≤ 0.05; not significant (NS); *N* = 5; results representative of three experiments.

To assess the efficacy of MSC treatment *in vivo*, BALB/c mice were infected and treated as described in the methodology. In relation to the lesion growth, treatment with MSCs by intravenous or intralesional route did not induce protection when compared with the controls (Figure 2A). However, treatment with MSC by intravenous route increased the parasite load in the infected footpad in relation to control (PBS i.v.) and other groups (PBS i.l. and MSC i.l.) (Figure 2B). The treatment with MSC by intralesional route did not affect the parasite load in comparison with control (PBS i.v.) (Figure 2B). There is no significant difference between any groups in terms of popliteal lymph nodes parasite load (Figure 2C). These results show that the use of i.v. therapy with MSC aggravated the infection caused by *L. amazonensis* at the infection site.

### Evaluation of Regulatory T Cells in the Spleen

**Figure 3:**
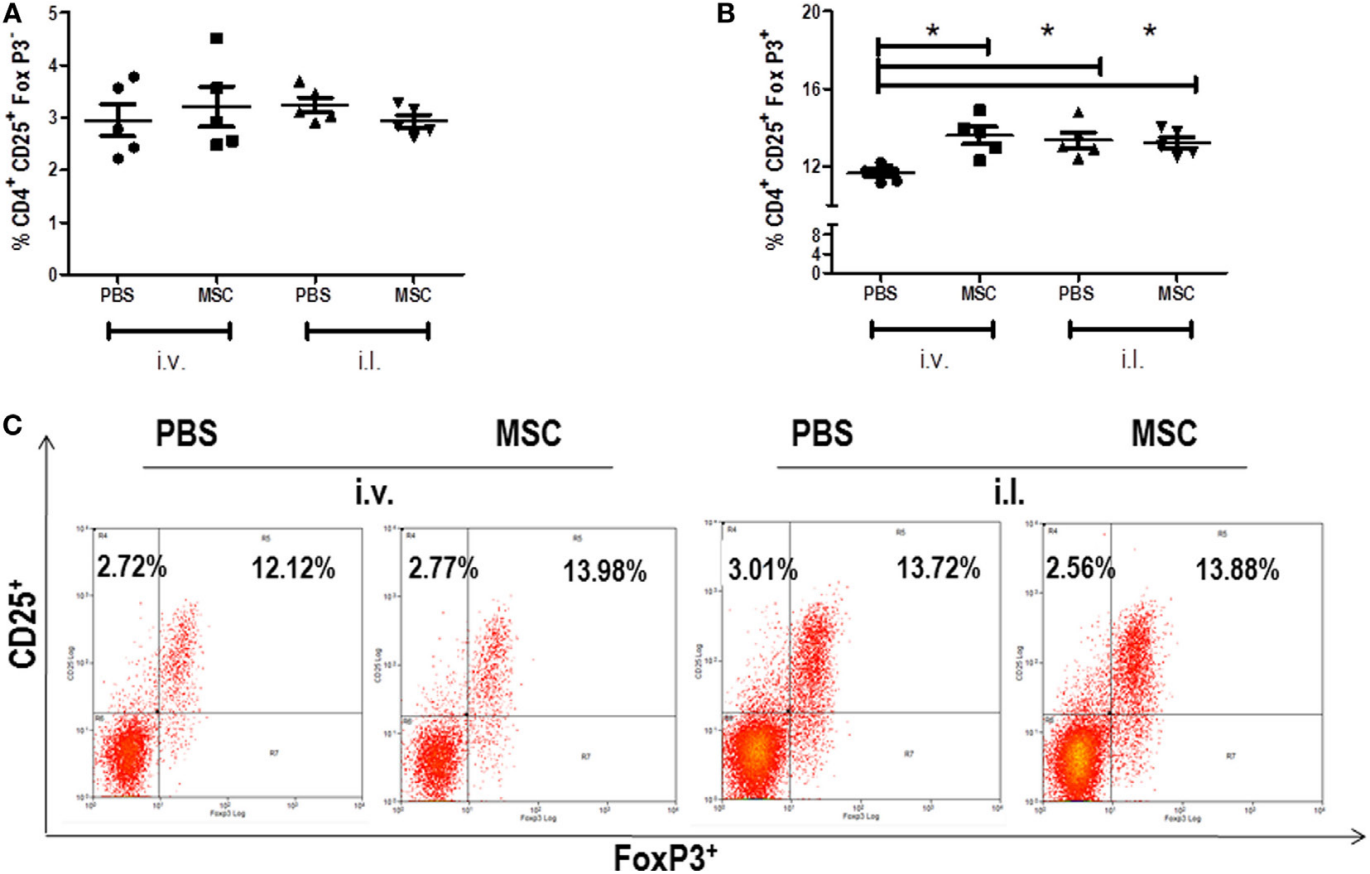
Percent of effector T cells and regulatory T cell in the spleen. The cells were collected from the spleen macerate. The percentage of CD4^+^ T cells in the spleens of the groups was evaluated through the CD4^+^ marker by flow cytometry (FACSCalibur BD), using antibody (BD), following the supplier’s instructions. CD4^+^ T cells, (CD4^+^ CD25^+^ FoxP3^−^) **(A)**, Treg (CD4^+^ CD25^+^ FoxP3^+^) **(B)** Dot plot of CD25 and FoxP3 staining gated on CD4^+^ spleen cells **(C)**. Data are presented as significant and ± Standard Deviation for each group. Representative graph of 2 experiments. *(*P* < 0.05) indicates significant statistical difference.

Based on the capacity of MSC therapy act systemically, we investigated the cell and cytokine profiles in the spleen. We evaluated the percentage of effector T cells (CD4^+^ CD25^+^ FoxP3^−^) and Treg cells (CD4^+^ CD25^+^ FoxP3^+^) by quantification of CD25^+^ and FoxP3^+^ markers in CD4^+^ T cells. In both groups, the percentages of effector T cells were similar (Figure 3A). However, there were increases of Treg cells in intravenous MSC treated animals when compared to PBS i.v. group, which was not observed when comparing MSC i.l. group to its control (PBS i.l.) (Figure 3B). It could be observed, by comparing intravenous versus intralesional treatment, that intralesional groups PBS i.l. and MSC i.l. showed an increase in relation to PBS i.v.; however, no difference was noted when compared with MSC i.v. These results suggest that Treg alone was not associated to an increase of parasite load.

### Treatment with MSC by i.v. Route Increased the Percentage of IL-10 Producing T Cells

**Figure 4:**
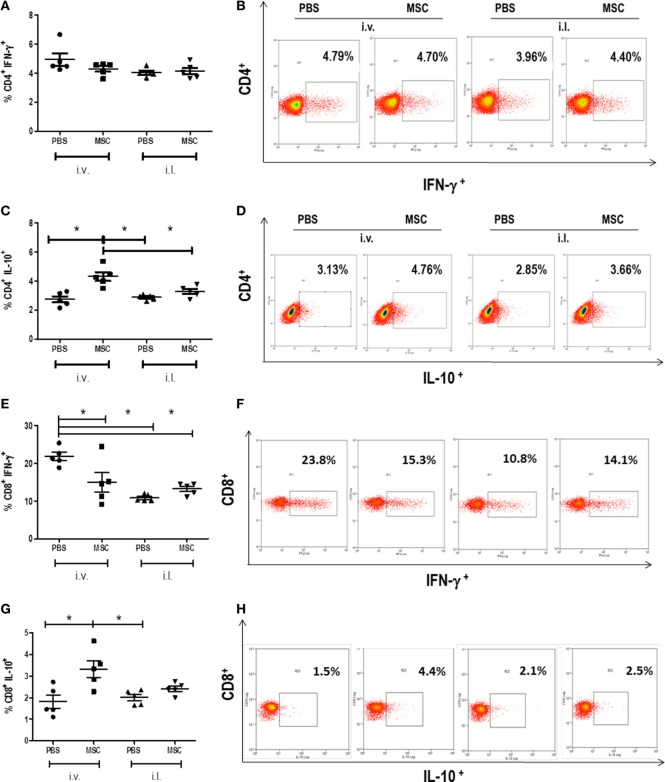
Detection of cytokines produced by CD4^+^ T cells and CD8^+^ T cells in the spleen. The cells were collected from the spleen macerate. Cells were analyzed by flow cytometry (FACS CALIBUR BD) for expression of CD4^+^ lymphocytes cytokine-producers of IFN-γ **(A)** and IL-10 **(C)** and of CD8^+^ lymphocytes cytokine-producers of IFN-γ **(E)** and IL-10 **(G)**. Results were shown as percentage of positive cells for these markers. Dot plot of IFN-γ **(B)** and IL-10 **(D)** staining gated on CD4^+^ spleen cells. Dot plot of IFN-γ **(F)** and IL-10 **(H)** staining gated on CD8^+^ spleen cells. Data are presented as significant and ± Standard Deviation for each group. Representative graph of 2 experiments. *(*P* < 0.05) indicates statistically significant difference.

We evaluated the percentage of IFN-γ and IL-10 produced by CD4^+^ and CD8^+^ T cells in the spleen. We observed the increase of CD4^+^ and CD8^+^ T cells producing IL-10 in mice treated with MSC i.v. in comparison to PBS i.v., but not with MSC i.l. in comparison to PBS i.l. (Figures 4C,G). When we compared intralesional versus intravenous treatment, it was observed that IL-10 produced by T CD4^+^ in MSC i.v. was increased in relation to PBS i.l. and MSC i.l., and no difference was observed between PBS i.v. against PBS i.l. and MSC i.l. The production of IL-10 by TCD8^+^ was increased in MSC i.v. in comparison to PBS i.l., but not in relation to MSC i.l. At the same time, we observed a reduction of IFN-γ produced by TCD8^+^, but not by CD4^+^ T cells, in animals treated with MSC i.v. in comparison to PBS i.v. No difference was observed on mice treated with MSC i.l. (Figure 4). No difference was observed in terms of production of IFN-γ by TCD4^+^ between the intravenous and intralesional treatments. However, in the production of IFN-γ by TCD8^+^, we observed a decrease of production of PBS i.l. and MSC i.l. in comparison to PBS i.v. and no difference was observed when compared MSC i.v. against PBS i.l. and MSC i.l. Altogether, the production of IL-10 by T CD4^+^ and T CD8^+^ cells in the group treated with MSC by i.v. is probably the major mechanism associated to increase the susceptibility to infection.

### Evaluation of Cytokine Production in the Footpad

**Figure 5:**
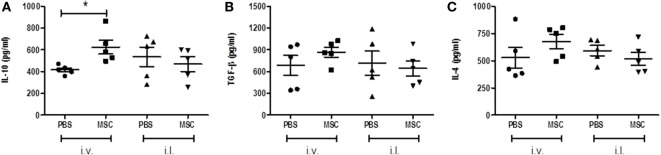
Analysis of cytokines in footpad. After euthanasia, the infected footpad of each animal was removed and a homogenate of the lesion was obtained by manual maceration. The concentrations of IL-10 **(A)**, TGF-β **(B)**, and IL-4 **(C)**. DC cytokines present in the homogenate’s supernatants were determined by enzyme-linked immunosorbent assay using a BD commercial kits, according to the manufacturer’s instructions. Expressed in picograms per milligram of tissue. * (*P* < 0.05); (#) Not determined; *N* = 5; representative result of two experiments.

After observing the deleterious effect of MSC by the i.v. route and the increased production of IL-10 by CD4^+^ T cells and CD8^+^ T cells, we evaluated the profile of pathogenic cytokines in the lesion by ELISA on footpad homogenate. There was a significant increase in IL-10 (Figure 5A) in the groups treated with MSC by i.v. when compared to the group treated with PBS by i.v., a fact that was not observed in the MSC i.l. group in relation to its controls (PBS i.l.). No difference was noted between intralesional and intravenous treatments (Figure 5A). This result suggests the importance of IL-10 only in i.v. treatment. There were no meaningful differences between the treated groups when compared to their controls in terms of concentration of TGF-β (Figure 5B) and IL-4 (Figure 5C).

## Discussion

*Leishmania amazonensis* is a member of the *Leishmania mexicana* complex and an etiological agent of diffuse cutaneous leishmaniasis (DCL) (21). Patients with DCL have some clinical signs, such a low rate of T-cell stimulation *in vitro* and fail in skin hypersensitivity tests using *Leishmania* antigens (negative Montenegro reaction), although some patients continue to respond to other antigens, such as tuberculin and lepromin (21). According to the Brazilian Ministry of Health, since 2005, the presence of *L. amazonensis* has been demonstrated in all Brazil regions (2), thus raising the concern about this infection.

As this form of the disease is refractory to conventional treatment, the search for new therapies is essential to fight this pathology. Due to the characteristics described above, cellular therapy would be an alternative for tackling this disease and to reestablish the homeostasis of the infected tissues. Several studies have shown that MSC cells contribute in a paracrine way in tissue remodeling and control of inflammation (22). This is observed in parasitic diseases like *T. cruzi* (11, 12, 23) and *Schistosoma japonicum* (24). In some cases, it may have a direct effect, such as in the elimination of bacteria by inducing antimicrobial molecules, such aslipocalin 2, in the treatment of pneumonia caused by *Escherichia coli* infection (25), or by increasing (26) or employing IFN-gamma-activated MSCs that induce GTPases and guanylate-binding proteins that exhibit anti-parasitic action against *Toxoplasma gondii* and *Neospora caninum* (27).

In our study, we expected that the MSC treatment would reduce the parasite load and lesion size; however, treatments with MSC were not effective in cutaneous leishmaniasis models caused by *L. amazonensis* in BALB/c mice. The treatment with MSC by the intralesional and intravenous routes did not affect the lesion development (Figure 2A). Likewise, the i.l. injection of MSC did not change the number of parasites. However, treatment with MSC i.v. unexpectedly increased the parasite load (Figure 2B), which may suggest a systemic mechanism for this immunomodulation. In the spleen, we observed an increase of TCD8^+^ and TCD4^+^ cells secreting IL-10. The production of IL-10 by T cells is a possible mechanism associated to the pathogenesis of intravenous treatment with MSC that is not observed in the intralesional treatment.

The production of cytokines in leishmaniasis plays a key role in maintaining susceptibility or resistance to disease, with cytokines IL-10, IL-4, TGF-β and IFN-γ being more relevant. The IL-10, in our infection model, has been associated to pathogenesis. IL-10 plays a role in limiting IFN-γ production by CD4^+^ and NK T cells during *Leishmania* infection (28, 29). IL-10 has been able to suppress NO production and leishmanicidal activities in the macrophages, leading to the suppression of Th1 response, resulting in the continuous progression of infection in susceptible mice and preventing the elimination of *Leishmania* (sterile cure) in resistant mice (29). In mice infected with *L. amazonensis*, IL-10 partially contributes to the generation of poor immune response (30–32), which is the main factor that increases susceptibility in mice co-infected with phlebotomine saliva (33). Recently, it was demonstrated that IL-10 is exclusively produced by T cells and not by macrophage, and granulocytes are associated to pathogenesis in infections caused by *L. mexicana* (34). The literature indicates the pathogenic role of IL-10 production by T cells, similar what has been observed in our model in the group treated with MSC by i.v. route.

Padigel et al. (35) showed that IL-10 deficient BALB/c presented greater resistance to infection with *L. mexicana* and *L. amazonensis*. Increased resistance in IL-10^−/−^ mice is related to the production of IFN-γ and NO, although their high production does not negatively regulate the development of a Th2 response and is observed by the continuous production of IL-4. In these animals (IL-10^−/−^) anti-IL-4 monoclonal antibodies were used. However, this treatment did not promote resolution of the infection, which did not evidence a relation of IL-4 with the pathogenesis of the disease in this model. Regarding the levels of IL-4 and TGF-β in our study, we did not obtain significant differences among treated groups, which suggest the lack of pathogenesis modulation by IL-4 and TGF-β. The high concentration of IL-10 (Figure 5A) found in the footpad from mice treated with MCS by i.v. route might be negatively modulating the activation of the macrophages capable of eliminating *Leishmania*, which increases the parasite load (Figure 2B).

Previous observations have shown that parasite-specific Th1 cells promoted protective immunity to *Leishmania* ssp. infection (36). In addition, in the *L. amazonensis* infection, the role of IFN-γ *in vivo* was demonstrated as being essential to the control of lesion growth and parasite load in the chronic phase (37). Studies have shown that TCD8^+^ has a protective role in *L. amazonensis* infection, with effector mechanisms associated with TCD8^+^ producing INF-γ and perforin, to provide a protective immune response against parasites (38). The decrease of interferon-gamma production by TCD8^+^ in the MSC therapy by i.v. route associated with the increase of IL-10 could be directly related with the increase of parasite load and associated to pathogenesis. We observed a decrease of IFN-γ by TCD8^+^ cell in intralesional groups (PBS i.l. and MSC i.l.); however, without the increase of IL-10 level, the parasite load did not rise. The concomitant increase of IL-10 and decrease of IFN-γ may be the main factor of failure of our therapy.

Tregs are crucial maintaining the homeostasis and regulatory T cells do not serve only to prevent autoimmune diseases, but they can also generate suppressive immune responses that contribute to non-destruction of tissue infected by infectious agents (39, 40). In *L. amazonensis* infection, Treg cells are important in the control of lesion and parasite load in resistant mice model using C57BL6 (41); however, in BALB/c, the importance of Tregs in *L. amazonensis* is unknown. Ji et al. (41) showed in C57BL/6 that treatment with neutralizing murine Ab against TGF-β1 and IL-10 did not eliminate the effect of Treg cells *in vitro*, demonstrating the role of cell–cell interaction molecules and, at the same time, suggesting that IL-10 does not contribute to the protective effect.

Corroborating with the literature (39, 40), we observed that the groups treated with intravenous MSC presented an increase of regulatory T cells (CD4^+^ CD25^+^ FoxP3^+^) in the spleen (Figure 3B) in relation to their control (PBS i.v.). One of our hypotheses is that MSCs could induce regulatory T cells and control the pathology in *Leishmania* infection. MSC was able to perform the induction of Treg cells in the group treated with MSC by i.v. route in comparison with the control (PBS i.v.), but it did not protect against the lesion as expected based on C57BL6 mice model. The increase of Treg did not affect growth lesion, suggesting that lesion and Treg on BALB/c mice may not be related. Furthermore, Treg cells were not contra-protective either in our model, since the increase of Treg in intralesional groups (PBS i.l. and MSC i.l.) did not result in the increase of parasite load in the intralesional groups. Interesting, the groups that presented an increase of Treg (MSC i.v., PBS i.l., and MSC i.l.) showed a decrease of TCD8^+^ producing IFN-g, which suggests a possible function for Treg cells in BALB/c mice.

Mesenchymal stromal cell therapy through different administration routes (MSC i.l. and MSC i.v.) when compared with controls (PBS i.l. and i.v.) did not show protective effect against *L. amazonensis* infection. Therefore, more studies must be conducted to find therapies that are effective against leishmaniasis due to the toxicity of the current pharmacological treatments used in the clinical setting. Besides, as it has been reported, there are significant concerns in that the application of mesenchymal stem cell may suppress antimicrobial immunity with increased risk of infection (42).

Further studies are necessary to a complete understanding of the treatment using MSC against leishmaniasis. First, experimental cutaneous leishmaniasis was induced by *L. amazonensis* in BALB/c mice. Therefore, these results cannot be extrapolated to other leishmaniasis models, mouse strain or directly to the clinical scenario. Second, two doses of (10^5^) have been used and the effects of MSCs were evaluated 5 days after the second administration. Based on pilot studies, higher number of MSCs may increase the risk of embolism. Therefore, we decided to administer this dose twice. Nonetheless, in future studies with different doses, different ranges between MSC administration and analysis at earlier and later time points would be interesting. Third, MSCs from different sources exhibited distinct effects (43, 44); however, only MSC from bone marrow was used. Further studies are required using different MSC sources.

## Conclusion

In the current murine model of cutaneous disease induced by *L. amazonensis*, bone marrow MSC therapy, in comparison with PBS, did not result in any significant difference in lesion progression, regardless of the administration route. Intravenous, but not intralesional administration of MSCs, increased the parasite load. BM-MSC therapy is not effective as a treatment against murine leishmaniasis.

## Author Contributions

Conceived and designed the experiments: HG and PR. Performed the experiments: JCP, TR, JS, JK, JESP, AF-M, MM, and LC. Analyzed data: JCP, TR, and JS. Scientific discussion: TR, JS, JCP, SC, DG, BD, PR, and HG. Contributed reagents/materials/analysis tools: HG, PR, and BD. Wrote the paper: TR, JS, JCP, SC, DG, BD, PR, and HG.

## Conflict of Interest Statement

The authors declare that the research was conducted in the absence of any commercial or financial relationships that could be construed as a potential conflict of interest.

## Supplementary Material

Figure S1 Phenotypic characterization of MSC. Bone marrow cells were obtained from the femur and tibia of BALB/c mice and maintained in culture until the fourth passage. About 1 × 10^6^ cells were characterized as MSCs according to the International Society of Cellular Consensus Therapy. Characterization of MSC cells was done using BD flow cytometry FACSCalibur. **(A)** CD 44 (Pecy5); **(B)** CD 49 (PE); **(C)** Sca-1 (APC); **(D)** CD 11b (FITC); **(E)** CD 45 (PE); **(F)** CD 34 (Effluor 660); and **(G)** MSC profile.

## References

[1] Silveira FT Lainson R De Castro Gomes CM Laurenti MD Corbett CE. Immunopathogenic competences of *Leishmania (V.) braziliensis* and *L. (L.)amazonensis* in American cutaneous leishmaniasis. Parasite Immunol (2009) 31(8):423–31. 10.1111/j.1365-3024.2009.01116.x 19646206

[2] Ministério da Saúde, Secretaria de Vigilância em Saúde, Departamento de vigilância Epidemiológica. Manual de vigilância de Leishmaniose Tegumentar Americana. 2 ed Série A. Normas e Manuais Técnicos. Brasília: Editora do Ministério da Saúde (2007). 182 p. Available from: http://bvsms.saude.gov.br/bvs/publicacoes/manual_vigilancia_leishmaniose_2ed.pdf

[3] Barral A Pedral-Sampaio D Grimaldi Júnior G Momen H McMahon-Pratt D Ribeiro de Jesus A Leishmaniasis in Bahia, Brazil: evidence that *Leishmania amazonensis* produces a wide spectrum of clinical disease. Am J Trop Med Hyg (1991) 44(5):536–46. 10.4269/ajtmh.1991.44.536 2063957

[4] Soong L. Subversion and utilization of host innate defense by *Leishmania amazonensis*. Front Immunol (2012) 3:58. 10.3389/fimmu.2012.00058 22566939 PMC3342205

[5] Pereira BA Alves CR. Immunological characteristics of experimental murine infection with *Leishmania (Leishmania) amazonensis*. Vet Parasitol (2008) 158(4):239–55. 10.1016/j.vetpar.2008.09.015 18922635

[6] Abreu SC Weiss DJ Rocco PR. Extracellular vesicles derived from mesenchymal stromal cells: a therapeutic option in respiratory diseases? Stem Cell Res Ther (2016) 7(1):53. 10.1186/s13287-016-0317-0 27075363 PMC4831172

[7] Cruz FF Weiss DJ Rocco PR. Prospects and progress in cell therapy for acute respiratory distress syndrome. Expert Opin Biol Ther (2016) 16(11):1353–60. 10.1080/14712598.2016.1218845 27487721

[8] Orlic D Kajstura J Chimenti S Jakoniuk I Anderson SM Li B Bone marrow cells regenerate infarcted myocardium. Nature (2001) 410(6829):701–5. 10.1038/35070587 11287958

[9] Orlic D Kajstura J Chimenti S Limana F Jakoniuk I Quaini F Mobilized bone marrow cells repair the infarcted heart, improving function and survival. Proc Natl Acad Sci U S A (2001) 98(18):10344–9. 10.1073/pnas.181177898 11504914 PMC56963

[10] Soares MB Lima RS Rocha LL Takyia CM Pontes-De-Carvalho L De Carvalho AC Transplanted bone marrow cells repair heart tissue and reduce myocarditis in chronic chagasic mice. Am J Pathol (2004) 164(2):441–7. 10.1016/S0002-9440(10)63134-3 14742250 PMC1602272

[11] Goldenberg RC Jelicks LA Fortes FS Weiss LM Rocha LL Zhao D Bone marrow cell therapy ameliorates and reverses chagasic cardiomyopathy in a mouse model. J Infect Dis (2008) 197(4):544–7. 10.1086/526793 18237267 PMC2671018

[12] Mello DB Ramos IP Mesquita FC Brasil GV Rocha NN Takiya CM Adipose tissue-derived mesenchymal stromal cells protect mice infected with *Trypanosoma cruzi* from cardiac damage through modulation of anti-parasite immunity. PLoS Negl Trop Dis (2015) 9(8):e0003945. 10.1371/journal.pntd.0003945 26248209 PMC4527728

[13] Soong L Chang CH Sun J Longley BJ Jr Ruddle NH Flavell RA Role of CD4+ T cells in pathogenesis associated with *Leishmania amazonensis* infection. J Immunol (1997) 158(11):5374–83. 9164958

[14] Dameshghi S Zavaran-Hosseini A Soudi S Shirazi FJ Nojehdehi S Hashemi SM. Mesenchymal stem cells alter macrophage immune responses to *Leishmania major* infection in both susceptible and resistance mice. Immunol Lett (2016) 170:15–26. 10.1016/j.imlet.2015.12.002 26703818

[15] Khosrowpour Z Hashemi SM Mohammadi-Yeganeh S Soudi S Pretreatment of mesenchymal stem cells with *Leishmania major* soluble antigens induce anti-inflammatory properties in mouse peritoneal macrophages. J Cell Biochem (2017) 118(9):2764–79. 10.1002/jcb.25926 28176354

[16] Dominici M Blanc LEK Mueller I Slaper-Cortenbach I Marini F Krause D Minimal criteria for defining multipotent mesenchymal stromal cells. The International society for cellular therapy position statement. Cytotherapy (2006) 8:315–7. 10.1080/14653240600855905 16923606

[17] Nombela-Arrieta C Ritz J Silberstein LE. The elusive nature and function of mesenchymal stem cells. Nat Rev Mol Cell Biol (2011) 12(2):126–31. 10.1038/nrm3049 21253000 PMC3346289

[18] Phinney DG Kopen G Isaacson RL Prockop DJ. Plastic adherent stromal cells from the bone marrow of commonly used strains of inbred mice: variations in yield, growth, and differentiation. J Cell Biochem (1999) 72(4):570–85. 10.1002/(SICI)1097-4644(19990315)72:4<570::AID-JCB12>3.3.CO;2-N 10022616

[19] Meirelles LS Nardi NB. Murine marrow-derived mesenchymal stem cell: isolation, *in vitro* expansion, and characterization. Br J Haematol (2003) 123(4):702–11. 10.1046/j.1365-2141.2003.04669.x 14616976

[20] de Matos Guedes HL da Silva Costa BL Chaves SP de Oliveira Gomes DC Nosanchuk JD De Simone SG Intranasal vaccination with extracellular serine proteases of *Leishmania amazonensis* confers protective immunity to BALB/c mice against infection. Parasit Vectors (2014) 7:448. 10.1186/1756-3305-7-448 25239157 PMC4261548

[21] Silveira FT Lainson R Corbett CE. Clinical and immunopathological spectrum of American cutaneous leishmaniasis with special reference to the disease in Amazonian Brazil: a review. Mem Inst Oswaldo Cruz (2004) 99:239–51. 10.1590/S0074-02762004000300001 15273794

[22] Duffy MM Ritter T Ceredig R Griffin MD. Mesenchymal stem cell effects on T-cell effector pathways. Stem Cell Res Ther (2011) 2(4):34. 10.1186/scrt75 21861858 PMC3219065

[23] Jasmin Jelicks LA Koba W Tanowitz HB Mendez-Otero R Campos De Carvalho AC Mesenchymal bone marrow cell therapy in a mouse model of Chagas disease. Where do the cells go? PLoS Negl Trop Dis (2012) 6(12):e1971 10.1371/journal.pntd.0001971 23272265 PMC3521704

[24] Xu H Qian H Zhu W Zhang X Yan Y Mao F Mesenchymal stem cells relieve fibrosis of *Schistosoma japonicum*-induced mouse liver injury. Exp Biol Med (2012) 237(5):585–92. 10.1258/ebm.2012.011362 22678013

[25] Gupta N Krasnodembskaya A Kapetanaki M Mouded M Tan X Serikov V Mesenchymal stem cells enhance survival and bacterial clearance in murine *Escherichia coli* pneumonia. Thorax (2012) 67(6):533–9. 10.1136/thoraxjnl-2011-201176 22250097 PMC3358432

[26] Mei SH Haitsma JJ Dos Santos CC Deng Y Lai PF Slutsky AS Mesenchymal stem cells reduce inflammation while enhancing bacterial clearance and improving survival in sepsis. Am J Respir Crit Care Med (2010) 182(8):1047–57. 10.1164/rccm.201001-0010OC 20558630

[27] Spekker K Leineweber M Degrandi D Ince V Brunder S Schmidt SK Antimicrobial effects of murine mesenchymal stromal cells directed against *Toxoplasma gondii* and *Neospora caninum*: role of immunity-related GTPases (IRGs) and guanylate-binding proteins (GBPs). Med Microbiol Immunol (2013) 202(3):197–206. 10.1007/s00430-012-0281-y 23269418

[28] Sakaguchi S Regulatory T cells: mediating compromises between host and parasite. Nat Immunol (2003) 4:10–1. 10.1038/ni0103-10 12496970

[29] Rocha PN Almeida RP Bacellar O De Jesus AR Filho DC Filho AC Down-regulation of Th1 type of response in early human American cutaneous leishmaniasis. J Infect Dis (1999) 180(5):1731–4. 10.1086/315071 10515843

[30] Mcmahon-Pratt D Alexander J Does the *Leishmania major* paradigm of pathogenesis and protection hold for new world cutaneous leishmaniasis or the visceral disease? Immunol Rev (2004) 201:206–24. 10.1111/j.0105-2896.2004.00190.x 15361243

[31] Jones DE Buxbaum LU Scott P. IL-4-independent inhibition of IL-12 responsiveness during *Leishmania amazonensis* infection. J Immunol (2000) 165:364–72. 10.4049/jimmunol.165.1.364 10861073

[32] Ji J Sun J Soong L. Impaired expression of inflammatory cytokines and chemokines at early stages of infection with *Leishmania amazonensis*. Infect Immun (2003) 71:4278–88. 10.1128/IAI.71.8.4278-4288.2003 12874303 PMC166010

[33] Norsworthy NB Sun J Elnaiem D Lanzaro G Soong L. Sand fly saliva enhances *Leishmania amazonensis* infection by modulating interleukin-10 production. Infect Immun (2004) 72(3):1240–7. 10.1128/IAI.72.3.1240-1247.2004 14977924 PMC356033

[34] Buxbaum LU Interleukin-10 from T cells, but not macrophages and granulocytes, is required for chronic disease in *Leishmania mexicana* infection. Infect Immun (2015) 83(4):1366–71. 10.1128/IAI.02909-14 25605773 PMC4363430

[35] Padigel UM Alexander J Farrell JP The role of interleukin-10 in susceptibility of BALB/c mice to infection with *Leishmania mexicana* and *Leishmania amazonenses*. J Immunol (2003) 171:3705–10. 10.4049/jimmunol.171.7.3705 14500669

[36] Maspi N Abdoli A Ghaffarifar F. Pro- and anti-inflammatory cytokines in cutaneous leishmaniasis: a review. Pathog Glob Health (2016) 110(6):247–60. 10.1080/20477724.2016.1232042 27660895 PMC5070640

[37] Pinheiro RO Rossi-Bergmann B Interferon-gamma is required for the late but not early control of *Leishmania amazonensis* infection in C57Bl/6 mice. Mem Inst Oswaldo Cruz (2007) 102(1):79–82. 10.1590/S0074-02762007000100013 17294004

[38] Colmenares M Kima PE Samoff E Soong L Mcmahon-Pratt D. Perforin and gamma interferon are critical CD8^+^ T-cell-mediated responses in vaccine-induced immunity against *Leishmania amazonensis* infection. Infect Immun (2003) 71(6):3172–82. 10.1128/IAI.71.6.3172-3182.2003 12761096 PMC155724

[39] Maccario R Podestà M Moretta A Cometa A Comoli P Montagna D Interaction of human mesenchymal stem cells with cells involved in alloantigen-specific immune response favors the differentiation of CD4^+^T-cell subsets expressing a regulatory/suppressive phenotype. Haematologica (2005) 90:516–25. 15820948

[40] Di Ianni M Del Papa B De Ioanni M Moretti L Bonifacio E Cecchini D Mesenchymal cells recruit and regulate T regulatory cells. Exp Hematol (2008) 36:309–18. 10.1016/j.exphem.2007.11.007 18279718

[41] Ji J Masterson J Sun J Soong L. CD4+CD25+ regulatory T cells restrain pathogenic responses during *Leishmania amazonensis* infection. J Immunol (2005) 174(11):7147–53. 10.4049/jimmunol.174.11.7147 15905558 PMC2812412

[42] Lombardo E van der Poll T DelaRosa O Dalemans W. Mesenchymal stem cells as a therapeutic tool to treat sepsis. World J Stem Cells (2015) 7(2):368–79. 10.4252/wjsc.v7.i2.368 25815121 PMC4369493

[43] Antunes MA Abreu SC Cruz FF Teixeira AC Lopes-Pacheco M Bandeira E Effects of different mesenchymal stromal cell sources and delivery routes in experimental emphysema. Respir Res (2014) 15:118. 10.1186/s12931-014-0118-x 25272959 PMC4189723

[44] Abreu SC Antunes MA Xisto DG Cruz FF Branco VC Bandeira E Bone marrow, adipose, and lung tissue-derived murine mesenchymal stromal cells release different mediators and differentially affect airway and lung parenchyma in experimental asthma. Stem Cells Transl Med (2017) 6(6):1557–67. 10.1002/sctm.16-0398 28425576 PMC5689762

