# Supplementary figures and images for: Effects of Bone Marrow Mesenchymal Stromal Cell Therapy in Experimental Cutaneous Leishmaniasis in BALB/c Mice Induced by Leishmania amazonensis
Source: Front Immunol. 2017 Aug 10;8:893. doi: 10.3389/fimmu.2017.00893 (PMC5554126; doi:10.3389/fimmu.2017.00893)

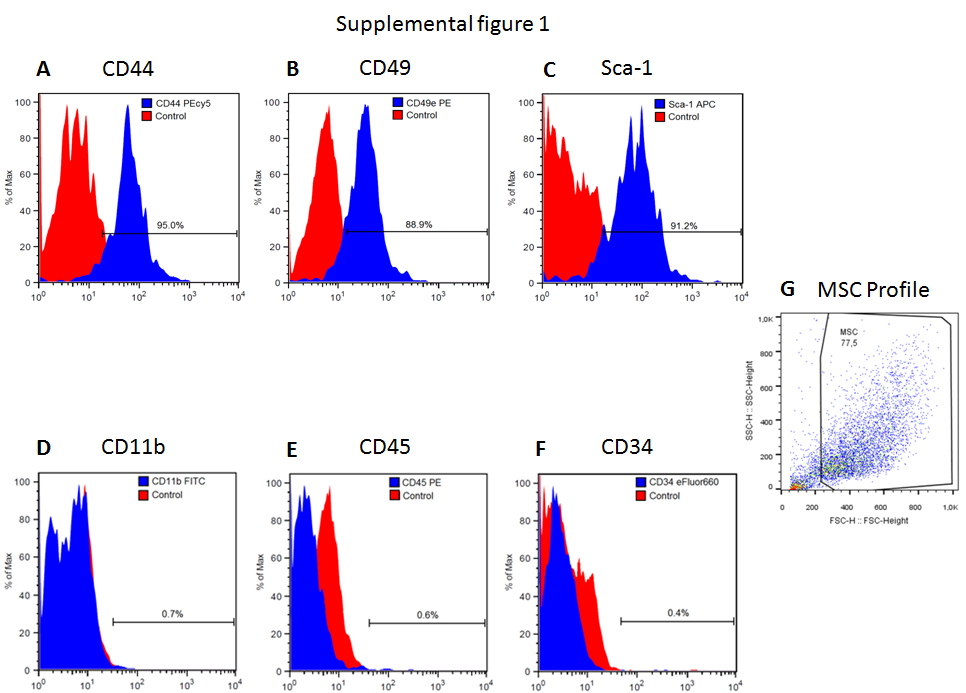

Supplement: Figure S1 — Phenotypic characterization of MSC. Bone marrow cells were obtained from the femur and tibia of BALB/c mice and maintained in culture until the fourth passage. About 1 × 10^6^ cells were characterized as MSCs according to the International Society of Cellular Consensus Therapy. Characterization of MSC cells was done using BD flow cytometry FACSCalibur. (A) CD 44 (Pecy5); (B) CD 49 (PE); (C) Sca-1 (APC); (D) CD 11b (FITC); (E) CD 45 (PE); (F) CD 34 (Effluor 660); and (G) MSC profile. [file Image_1.TIF]
